# Understanding the Lymph Node Microenvironment in Metastatic and Non-Metastatic Head and Neck Squamous Cell Carcinoma: A Systematic Review

**DOI:** 10.3390/jpm16090444

**Published:** 2026-08-24

**Authors:** Antoine Yanni, Géraldine Descamps, Fabrice Journe, Edward Boutremans, Isabelle Loeb, Sven Saussez, Didier Dequanter

**Affiliations:** 1 Department of Stomatology & Maxillofacial Surgery, Centre Hospitalier Universitaire Saint-Pierre, 322 Rue Haute, 1000 Brussels, Belgium; 2 Laboratory of Human Anatomy and Experimental Oncology, Faculty of Medicine, Research Institute for Health Sciences and Technology, University of Mons (UMONS), 8 Avenue du Champ de Mars, 7000 Mons, Belgium; 3 Laboratory of Oncology and Experimental Surgery, Jules Bordet Institute, 90 Rue Meylemeersch, 1070 Brussels, Belgium; 4 Department of Otorhinolaryngology-Head & Neck Surgery, Centre Hospitalier Universitaire Saint-Pierre, 322 Rue Haute, 1000 Brussels, Belgium

**Keywords:** lymph node microenvironment, innate immune system, adaptive immune system, head and neck squamous cell carcinoma, immunological pathways, immune cell distribution

## Abstract

**Background:** The immune landscape in head and neck squamous cell carcinoma (HNSCC) has been widely investigated. However, the crucial role played by the lymph node microenvironment in metastatic and non-metastatic HNSCC remains unknown. This systematic review aims to discuss the immunological crosstalk between the tumor and the nodal microenvironment and to describe the distribution of immune cells in metastatic and non-metastatic lymph nodes. **Methods:** A systematic review was conducted according to the PRISMA guidelines. PubMed, Scopus, and the Cochrane Library were searched for studies published between 1990 and 2025, with the final search performed in December 2025. Prospective and retrospective studies evaluating immune cell infiltration in metastatic and non-metastatic cervical lymph nodes were included, whereas studies focusing exclusively on non-cellular biomarkers and non-English publications were excluded. The risk of bias was assessed using the Newcastle–Ottawa Scale. The results were synthesized narratively, and no meta-analysis was performed. **Results:** The screening process identified 608 articles, of which 27 met our predefined inclusion criteria. These studies focused on macrophages, dendritic cells, neutrophils, natural killer cells, T helper cells, cytotoxic T cells, regulatory T cells, B cells, total lymphocytes, and surface markers. This systematic review provides a well-structured analysis of current knowledge on the impact of the innate and adaptive immune systems on the response against cancer cells and the recruitment of immune cells in lymph nodes. The most significant findings highlight the crucial role of antigen presentation in the antitumor response, particularly through the recruitment and activation of dendritic cells and subcapsular sinus macrophages in tumor-draining lymph nodes. It also presents in a fairly comprehensible manner that the density of mature dendritic cells, cytotoxic T cells, and B cells is higher in non-metastatic lymph nodes. **Discussion:** The lymph node microenvironment is highly enriched with immune cell infiltration, and their distribution between metastatic and non-metastatic lymph nodes can contribute to a better understanding of the underlying pathological processes. Particular attention should be given to the innate immune system cells and their implication in antigen presentation. Our findings suggest that identifying immunological profiles of lymph nodes may provide a rationale for treatment de-escalation protocols and raise the question of lymph node preservation in antitumor immune responses. However, the heterogeneity and bias assessment of the included studies warrant a cautious interpretation of these findings. Another important limitation is the limited number of studies comparing the immune microenvironment of primary tumors and lymph nodes.

## 1. Introduction

Head and neck squamous cell carcinoma (HNSCC) ranks as the sixth leading cause of cancer worldwide. The five-year survival rate remains poor and has not improved over the past 20 years [1,2]. In addition to primary treatments such as surgery, chemotherapy, and radiotherapy, immunotherapy has shown promising results. However, identifying the patients who will benefit from these novel approaches remains a major clinical challenge. There is limited information and significant uncertainty regarding disease progression [3,4]. The TNM system has been widely used but offers limited guidance in predicting treatment response or guiding therapeutic de-escalation [5,6].

**Figure 1:**
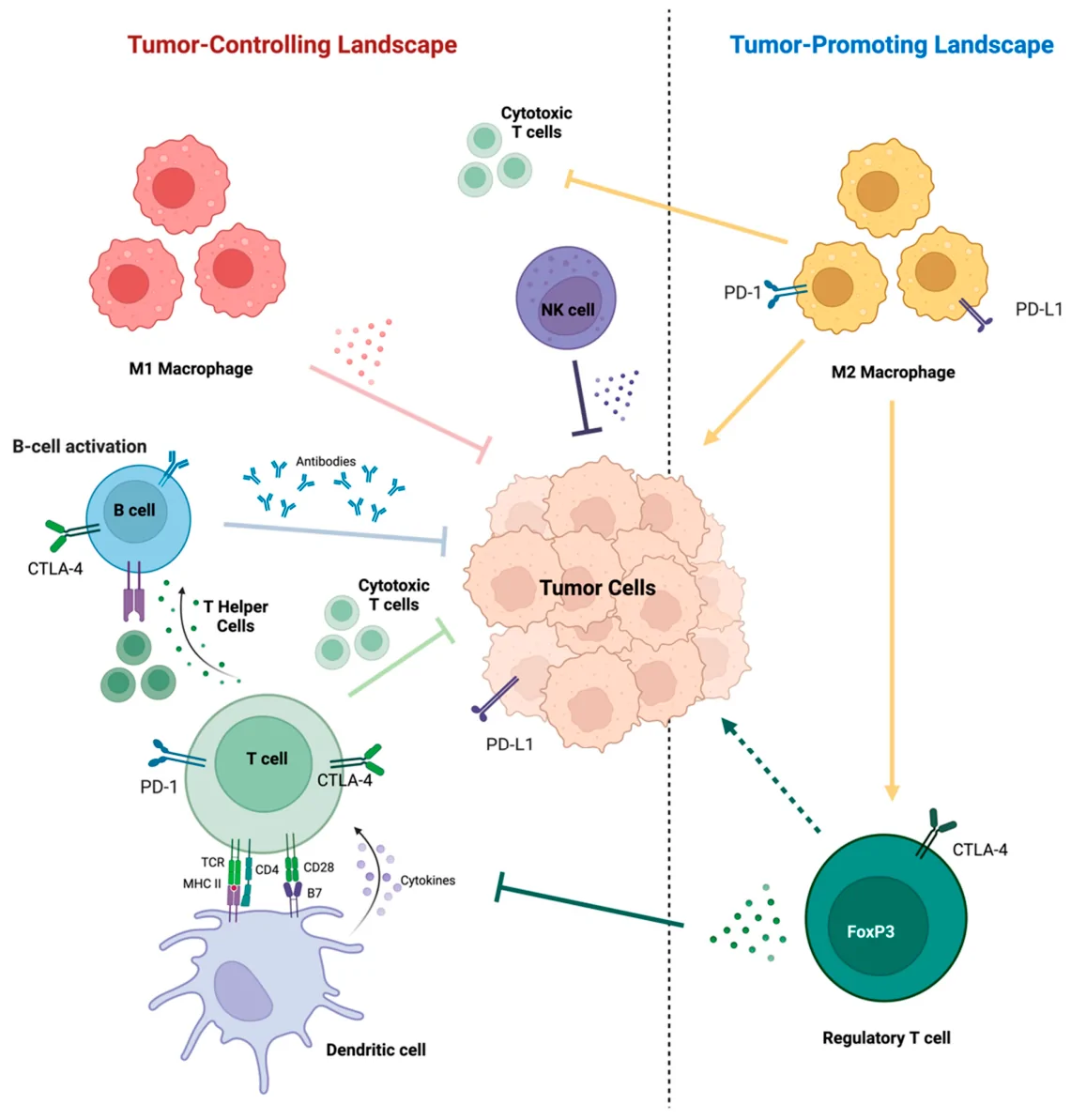
The double-edged sword of the tumor immune microenvironment. This theoretical overview of the immune landscape in the tumor environment presents the pro- and antitumoral functional orientations of immune cells.

There is increasing evidence suggesting that the immune microenvironment strongly influences disease prognosis and treatment response. Recent research on immune checkpoint inhibitors has proven that immune cells surrounding tumor cells can combat cancer [7,8,9]. The specific distribution of immune cells in the tumor microenvironment (TME) of HNSCC has been shown to significantly impact its favorable prognosis [10,11,12,13]. A theoretical overview of the tumor immune landscape is represented in Figure 1.

As lymph node metastases contribute to poor prognosis, understanding the underlying mechanisms is essential [14,15,16,17,18]. Crosstalk between cancer cells and immune cells occurs within both metastatic and healthy lymph nodes [14]. In this context, the lymph node microenvironment plays a crucial role and is believed to mediate the response against tumor cells in both primary tumors and lymph nodes [15,16]. The ambivalent role of lymph nodes—both initiating the antitumor response and facilitating the spread of tumor cells—remains unclear, and the underlying pathways require further investigation.

In various solid cancers, tumor cells precondition the lymph node microenvironment by releasing factors that modulate the microenvironment in tumor-draining lymph nodes (TDLNs) and form immunosuppressive pre-metastatic niches [17,18,19]. Antigen-presenting cells (APCs), such as dendritic cells (DCs), tumor-associated macrophages (TAMs), and monocytes, play a key role in this process. These APCs can capture tumor-derived antigens, either in the primary site or directly in lymph nodes, and present them to naïve T cells in the TDLNs, thus initiating the adaptive immune response [20,21,22]. Depending on the interaction of inhibitory or activating signals (e.g., CD80-86/CD28, MHC-TCR for stimulatory signals, and PD1, CTLA4, PDL1 for inhibitory signals), naïve T cells can activate the antitumor response [17,20,21].

In clinical practice, decisions regarding the surgical management of cervical lymph nodes are mainly based on histopathological and morphological findings. This approach does not consider the immunological specificity between lymph nodes. A better understanding of the lymph node microenvironment, especially in TDLNs, could help redefine personalized strategies and identify novel antitumoral therapies [15].

While the TME has been extensively studied in primary tumors, fewer data have been published on the immune environment within lymph nodes in HNSCC patients. This systematic review aims to discuss the immunological crosstalk between the TME and the nodal microenvironment and investigate the current knowledge of the immune system’s role in the development of lymph node metastases in HNSCC patients.

## 2. Materials and Methods

This study has not been registered in PROSPERO. As an alternative, it has been registered in INPLASY (number 202680066).

No generative artificial intelligence was used to create the scientific content of this manuscript.

The research question was defined through the PICOTS framework.

### 2.1. PICOTS Framework

#### 2.1.1. Population

The population consisted of patients diagnosed with HNSCC, with or without lymph node involvement. All tumor localizations of HNSCC, including oropharyngeal, laryngeal, hypopharyngeal, oral, and nasopharyngeal squamous cell carcinoma (SCC), were included.

#### 2.1.2. Intervention

Assessment of the immune microenvironment in cervical lymph nodes of patients with HNSCC, with or without lymph node involvement. Additionally, reports exploring the dialogue and immunological communication pathways between the primary tumor and lymph nodes.

#### 2.1.3. Comparison

Comparisons between metastatic and non-metastatic lymph nodes were considered whenever available. Studies comparing lymph node tissue with primary tumor tissue were also eligible.

#### 2.1.4. Outcomes

The primary outcome of this study was to investigate the infiltration of immune cells (including innate immune cells—macrophages, dendritic cells, neutrophils, and natural killer cells and adaptive immune cells—T helper cells, cytotoxic T cells, regulatory T cells, B cells, and lymphocytes) in the lymph nodes of HNSCC, both with and without lymph node metastasis. The secondary outcome aimed to examine tumor characteristics (TNM staging, histopathology) and survival outcomes, such as overall survival (OS) and disease-free survival (DFS), to evaluate the impact of the lymph node microenvironment on the prognosis of HNSCC.

#### 2.1.5. Timing

Extensive research was performed, and relevant articles published between 1990 and 2025 were eligible for inclusion.

#### 2.1.6. Setting

Prospective and retrospective clinical trials investigating the immune microenvironment in cervical lymph nodes of patients with HNSCC were included.

The following exclusion criteria were used:

(1) Studies that did not investigate the immune microenvironment of lymph nodes and cell infiltration in HNSCC.
(2) Studies focusing on indicators other than immune cells, such as cytokines, chemokines, gene expression, growth factors, endothelial cells, and fibroblasts.
(3) Papers that were not available or not written in English, as well as reviews, public communications, posters, personal opinions, or letters.

### 2.2. Search Strategy

**Figure 2:**
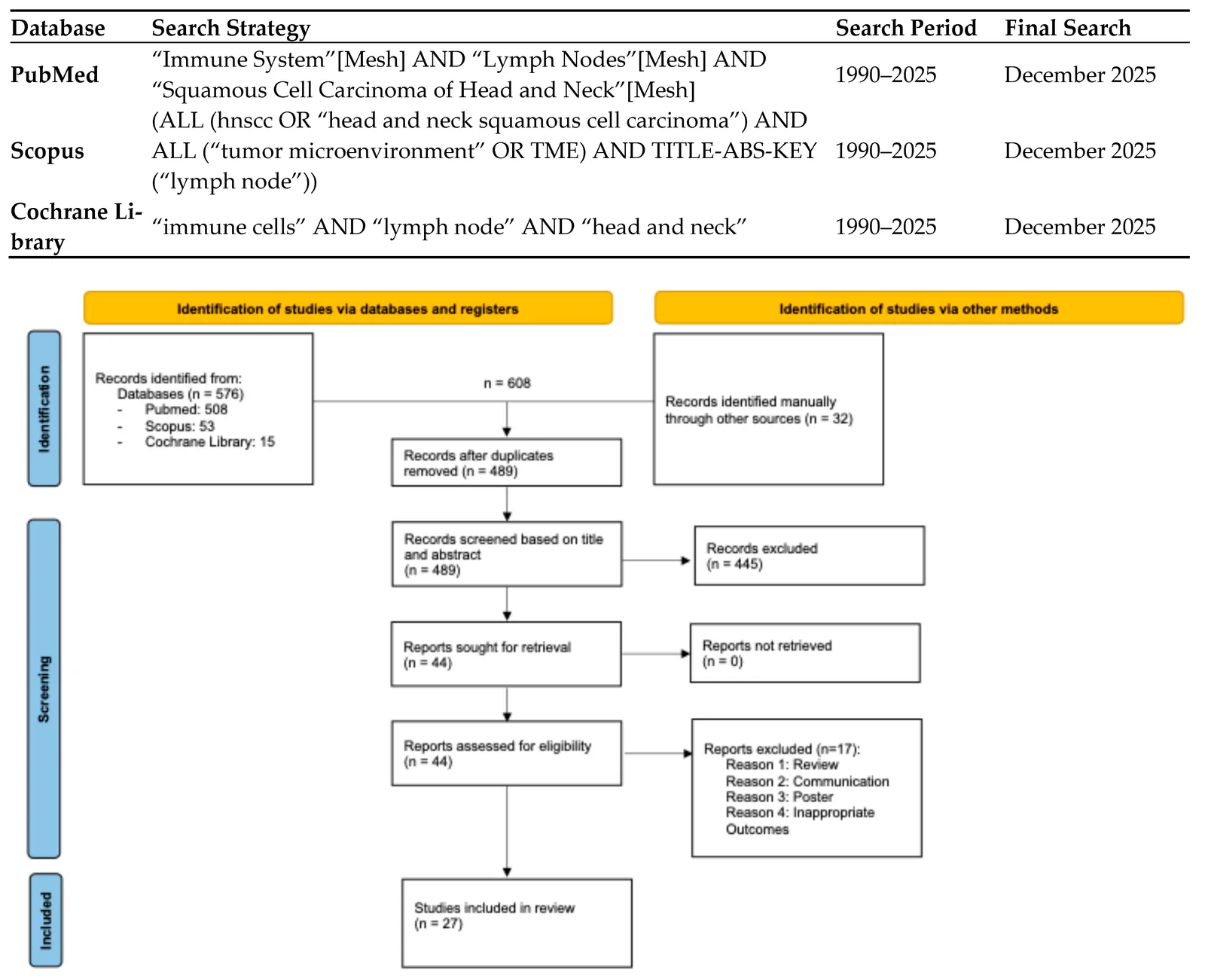
PRISMA flow diagram.

Two authors (AY and GD) conducted searches in PubMed, Scopus, the Cochrane Library, and manually through other sources to identify relevant articles that investigated the lymph node immune microenvironment in HNSCC. The research employed MESH terms such as “Macrophage Cells,” “Immunotherapy,” “Antigen Presenting Cells,” “Myeloid-Derived Suppressor Cells,” “Immune Environment,” “Plasma Cells,” “Dendritic Cells,” “Memory T Cells,” “Memory B Cells,” “T-Lymphocytes, Regulatory,” “Head and Neck Neoplasms,” “Lymph Nodes,” and “Prognosis.” Boolean operators (AND, OR) were used to combine these terms. The review adhered to the PRISMA checklist [23], and a flow diagram outlining the screening and selection process is presented in Figure 2. The complete search strategies are represented below.

### 2.3. Study Selection

Two independent authors (AY and GD) screened articles based on their titles and abstracts, considering the predefined inclusion criteria. Full-text reviews of selected articles were then conducted by the same authors to confirm their eligibility based on the previously described criteria. In cases of discrepancies, a third author (SS) was consulted to reach a consensus.

### 2.4. Data Collection

Data extracted from the selected articles were organized and stored in a Microsoft Excel sheet. The collected information included details related to the manuscript (author’s name, journal, publication date) and clinical data, such as TNM characteristics, tumor localization, HPV status, treatment approaches, study design, immune cells investigated, survival outcomes (OS, DFS), and the number of patients included.

AY and GD reviewed a total of 27 included articles, and a thorough verification process was performed to ensure the accuracy of the collected data.

### 2.5. Data Synthesis

Due to the heterogeneity of study designs, tumor subsites, immune markers, outcomes, and reporting, quantitative analysis of the data was not performed and considered inappropriate. Consequently, a meta-analysis was not feasible, and a narrative synthesis was conducted. Given these limitations, the certainty of the evidence was not formally assessed.

### 2.6. Risk of Bias in Individual Studies

We collected qualitative data on potential sources of bias in each study. The bias was analyzed using the recommended approach for assessing the risk of bias in non-randomized studies using the Newcastle–Ottawa Scale (NOS) tool. For each domain of bias, studies were classified into one of the following three categories: low, high, or unclear risk of bias.

## 3. Results

### 3.1. Article Selection and Characteristics

A total of 608 records were initially identified, including 576 records retrieved from database searches and 32 identified through additional sources. After duplicate removal, 489 records were screened. Ultimately, 27 studies fulfilled the predefined eligibility criteria and were included in the systematic review. All the included studies investigated the distribution and recruitment of immune cells within different lymph node compartments, including metastatic and non-metastatic lymph nodes. Among the investigations related to the innate and adaptive immune systems, the majority of studies focused their analyses on all subtypes of HNSCC patients. Only ten studies exclusively explored oral SCC, one study examined hypopharyngeal and laryngeal SCC, one study focused on oropharyngeal SCC, one study analyzed oral and oropharyngeal SCC, and 14 studies investigated all types of HNSCC. The NOS of the included studies (shown in Supplementary Table S1) ranged from four to nine, reflecting moderate heterogeneity in methodological quality. Most studies achieved satisfactory scores for the selection and comparability assessments. However, several studies were limited by the assessment of outcomes, follow-up length, and adequacy of follow-up cohorts. None of the studies was evaluated as being at high risk of bias or low quality.

The included studies adopted different definitions of lymph node status. However, metastatic and non-metastatic lymph nodes were used as the primary classification. Rubio et al. described pre-metastatic lymph nodes as lymph nodes exhibiting immunological changes induced by the tumor [24]. Sakakura et al. introduced the sentinel lymph nodes, defined as the first lymph nodes receiving drainage from the primary tumor [25]. Topf et al. focused on tumor-draining lymph nodes, representing the lymph nodes exposed to tumor-derived antigens [26]. 

### 3.2. Immune Cells

All studies directed their efforts toward analyzing immune cell recruitment, and studies solely evaluating the expression of cellular markers were rejected. A total of seven studies focused on macrophages [16,26,27,28,29,30,31], seven on dendritic cells (DCs) [22,25,30,31,32,33,34], two on neutrophils [35,36], two on natural killer cells (NK cells) [25,37], nine on T helper cells (Th cells) [24,27,31,38,39,40,41,42,43], 11 on cytotoxic T cells [11,16,24,25,26,27,28,31,38,41,42,43,44], four on regulatory T cells (Tregs) [22,27,44,45], six on B cells [24,30,31,43,44,46], six on lymphocytes without making any difference between subtypes [15,24,30,39,42,47], and four on surface markers [26,39,40,42] (Table 1). Table 2 summarizes innate immune cell infiltration in lymph nodes (macrophages, dendritic cells, neutrophils, and natural killer cells). Table 3 summarizes adaptive immune cell infiltration in lymph nodes (T helper cells, cytotoxic T cells, regulatory T cells, B cells, lymphocytes).

### 3.3. Neutrophils

The precise contribution of neutrophils to the antitumoral response is unresolved. Nevertheless, several authors have investigated their role in the lymph node microenvironment (Table 2). Only two papers have attempted to elucidate this. In 2021, Pylaeva et al. conducted a retrospective trial including 71 patients suffering from HNSCC. Neutrophil distribution in both primary tumors and lymph nodes was assessed by immunohistochemistry using the CD66b neutrophil cell marker. They reported that the number of neutrophils in B cell areas is comparable between metastatic and non-metastatic lymph nodes. However, a higher abundance of neutrophils in B cell follicles was associated with improved overall survival [35].

In a subsequent study, the same group confirmed these findings and provided additional results regarding the distribution of neutrophils. It was observed that the cells were mainly localized in sinuses, close to T cells, validating their role in T cell activation. Moreover, the protective effect of neutrophils on OS appeared to be effective only in non-metastatic patients. Conversely, in metastatic patients, neutrophil densities were associated with a decrease in survival. This suggests a switch from an anti- to a pro-tumor response during disease progression [36].

### 3.4. Natural Killer Cells

NK cells play a crucial role in the innate immune response against tumors. However, few research studies have reported the infiltration of these lymphocytes in lymph nodes from HNSCC patients (Table 2). Only two publications have examined the presence of NK cells in such lymph nodes. Sakakura et al. described that the infiltration of NK cells in both sentinel and non-sentinel lymph nodes is similar. Moreover, the comparison of metastatic and non-metastatic sentinel lymph nodes revealed no difference in terms of NK cell recruitment [34]. In contrast, Li et al. reported that NK cells were significantly more abundant in sentinel lymph nodes with occult metastases compared to tumor-free nodes [37]. 

### 3.5. Dendritic Cells

Goncalves et al. found that metastatic lymph nodes showed higher immature DC (CD1a DC) and lower mature DC (CD83 DC) values compared to non-metastatic lymph nodes (Table 2). No correlation with survival rates was performed [22]. Other authors claimed that there was no difference in mature and immature DC recruitment between these two compartments [25].

Li et al. reported that the overall density of dendritic cells was higher in non-metastatic lymph nodes than in metastatic lymph nodes, and that an increased expression of DCs was correlated with better OS [33].

Other authors contradicted these results, confirming that the total number of DCs is not affected by lymph node involvement [30,31,34]. According to O’Donnell et al., the only difference lies in the distribution of mature DCs, which are more abundant in non-metastatic lymph nodes of metastatic patients compared to lymph nodes of non-metastatic patients [34].

Kindt et al. provided somewhat contrasting findings, confirming that the number of DCs is higher in metastatic lymph nodes compared to non-metastatic nodes [32].

In an effort to understand and highlight the physiological pathways taken by DCs in the antitumor immune response, some studies have investigated the concentration gradient of the involved actors based on lymph node location. For example, O’Donnell et al. reported a decrease in the number of mature DCs proportional to the distance from the primary tumor, both in metastatic and non-metastatic patients [34]. However, these findings differ from other reports regarding immature DCs, as they do not show the same trend as mature DCs [25].

Overall, the findings we have gathered reveal a lower frequency of mature DCs in metastatic lymph nodes. Furthermore, DCs have shown an association with improved OS, and it appears that the recruitment of mature DCs is higher in tumor-draining lymph nodes, suggesting a substantial host investment in DC maturation and migration.

### 3.6. Macrophages

Jones et al. and Zhang et al. reported a higher density of macrophages in metastatic lymph nodes than in non-metastatic lymph nodes, although no differences in macrophage subtype were observed [16,30] (Table 2).

In HPV-associated oropharyngeal SCC, Tosi et al. found that lymph node metastases from HPV-positive patients had higher levels of CD68 and CD163 macrophages than those from HPV-negative patients. Higher macrophage densities were associated with improved DFS in HPV-positive patients, whereas increased M2 macrophage infiltration was associated with shorter DFS in HPV-negative patients [27] (Table 4).

In similar analyses performed in oral SCC, Wehrhan et al. reported comparable macrophage polarization in metastatic and non-metastatic lymph nodes [29]. In contrast, Wang et al. observed an increased proportion of M1 macrophages in metastatic lymph nodes [28]. Furthermore, in one of these studies, higher M2 macrophage infiltration was associated with lymphangioinvasion, perineural invasion, advanced T stage, and higher tumor grade [29].

The last study aimed at elucidating the role of subcapsular sinus macrophages (SSMs) in the antigen presentation process by examining the infiltration of CD169 macrophages. The results are presented further below in the chapter dedicated to surface markers [26].

In conclusion, the impact of macrophages in the lymph node microenvironment remains unclear. However, the findings support their involvement in antigen presentation and the antitumor response and suggest that M2 macrophages may contribute to tumor progression.

### 3.7. Regulatory T Cells

Several studies investigated Tregs infiltration in HNSCC lymph nodes and their association with clinical outcomes (Table 3). Goncalves et al., Pretscher et al., and Tosi et al. identified Tregs using FoxP3 immunohistochemistry [22,27,44]. Piersiala et al. were the only authors to consider Tregs, after flow cytometry, as FoxP3+/CD25^high^ [45].

Goncalves et al. conducted a study and reported significantly higher Treg counts in metastatic lymph nodes compared to non-metastatic lymph nodes [22]. Similarly, TDLNs exhibited a higher number of Tregs compared to non-TDLN [45]. In line with this, in HPV-positive patients, Tregs were even closer to metastatic tumor cells, further highlighting the dense immunological environment of these cancers [27] (Table 4).

Furthermore, other authors demonstrated that a higher number of Tregs was not significantly associated with improved DFS rates [44].

Upon reviewing these findings, the presence of Tregs in the immune response of HNSCC is suggested to be associated with metastatic nodes but does not seem to have an impact on prognosis.

### 3.8. T Helper Cells

Nine studies investigated Th cell infiltration in HNSCC lymph nodes (Table 3). Three studies reported no significant differences in Th cell density according to lymph node involvement [38,41,42]. In contrast, three studies suggested that Th cells are more abundant in healthy lymph nodes compared to pre-metastatic or metastatic lymph nodes [24,31,43].

When correlating these analyses with survival outcomes, lower densities of activated Th cells were associated with poorer OS and DFS. Similarly, this has been demonstrated in HPV-induced HNSCC patients, where higher densities of Th cells have been linked to improved DFS [27,39] (Table 4).

Analysis of Th cell surface markers revealed reduced CD161 marker (which expresses their migration capacity) expression in metastatic lymph nodes (especially in Th17 subsets) [40] (Table 3).

In conclusion, it emerges that Th cells may contribute to antitumor immunity but can also acquire essential migratory capabilities to fulfill their protective function.

### 3.9. Cytotoxic T Cells

A total of 11 studies explored CD8+ T cell infiltration (Table 3). Among them, nine papers compared the density of CD8+ T cells in metastatic and non-metastatic lymph nodes. Four reports concluded that the expression of CD8+ T cells is less frequent in metastatic lymph nodes [11,16,38,44], whereas only one report has shown the opposite [26]. The remaining studies found no significant difference between the two nodal compartments [31,41,42,43].

Tosi et al. reported that the density of CD8+ T cells is higher in metastatic lymph nodes and that the CD8+ T cells are closer to tumor cells (in HPV-induced HNSCC patients compared to HPV-negative HNSCC patients) (Table 4). In the same cohort, increased CD8+ T cell infiltration was associated with improved DFS [27].

Similarly, Pretscher et al. concluded that an increased number of CD8+ T cells is associated with improved DFS [44].

Wang et al. and Rahim et al. focused their research on the analysis of exhausted and pre-exhausted T cell subsets, and they revealed that the number of exhausted T cells is less frequent in non-metastatic lymph nodes (compared to the primary tumor) [43] and less frequent in metastatic lymph nodes (compared to non-metastatic lymph nodes) [28]. These results support a potential impact of exhausted T cells on tumor progression.

### 3.10. B Cells

Six studies examined the relationship between B cell infiltration and lymph node progression [24,30,31,43,44,46] (Table 3). Norouzian et al. reported similar B cell densities in metastatic and non-metastatic lymph nodes in laryngeal SCC. However, in tongue SCC, metastatic lymph nodes contained fewer B cells [46]. The significant drop in B cells in metastatic lymph nodes has also been proposed by other authors [30,43].

Other studies investigated B cell distribution between pre-metastatic, metastatic lymph nodes, and primary tumor, but the results remain inconsistent [24,30,44]. On one hand, some studies suggest that the number of B cells is higher in metastatic lymph nodes compared to primary tumors [30,44]. On the other hand, Rubio et al. observed a reduced number of B cells in control lymph nodes compared to pre-metastatic lymph nodes [24]. Only Pretscher et al. evaluated the prognostic value of B cells and reported an association between higher densities and improved DFS [44].

Rubio et al. also described the compartmental distribution of B cells in pre-metastatic lymph nodes. B cells (CD69−) were mainly located in the germinal center, T cells (CD69+) were predominant in the centro-germinal layer, B cells (CD69+) were concentrated in the mantle, and T cells (CD69−) were located outside the mantle [24].

### 3.11. Lymphocytes

To obtain an overall understanding of the immune landscape in HNSCC lymph nodes, numerous authors have examined the density of total, T, or B lymphocyte populations (Table 3). Rubio et al. reported higher lymphocyte densities in pre-metastatic lymph nodes than in control lymph nodes, suggesting early immunological changes induced by the primary tumor [24]. Regarding lymphocytes infiltrating the tumor, Kim et al. reported no significant differences in TIL densities between primary tumors and lymph node metastases [47].

### 3.12. Surface Markers

By identifying and analyzing the expression of activation surface markers, researchers can gain valuable insights into the specific cells and pathways involved in the immune response against tumor cells (Supplementary Table S2). Kagedal et al. reported that higher expression of activation surface markers (such as CD69, CD71, and HLA-DR) in tumor-infiltrated T cells reflects activation in the primary tumor [39]. Letessier et al. observed lower expression of CD56 and CD11b in lymph node lymphocytes than in peripheral blood lymphocytes, suggesting lower adhesion activity of lymph node lymphocytes [42].

Moreover, Topf et al. investigated this field by analyzing the quantification of CD169+, a surface marker implicated in antigen presentation and in the blockage of tumor exosome traffic in TDLNs. CD169+ SSM in TDLNs expressed higher levels in metastatic lymph nodes than in adjacent tumor-free lymph nodes [26].

## 4. Discussion

This systematic review emphasizes the current state of knowledge regarding the immune environment in metastatic and non-metastatic lymph nodes associated with HNSCC. We believe that comprehending the intricate interaction between the tumor and the lymph node can provide valuable insights [15,16].

A crucial aspect of the immune response against cancer cells is antigen presentation, which occurs initially and continues in diverse anatomical regions [15,20,21,26]. In order to mount an effective antitumor response, DCs must capture antigens, process them, and undergo an essential transformation from an immature state (CD1a+) to a mature state (CD83+) during their migration to the TDLN [48].

**Figure 3:**
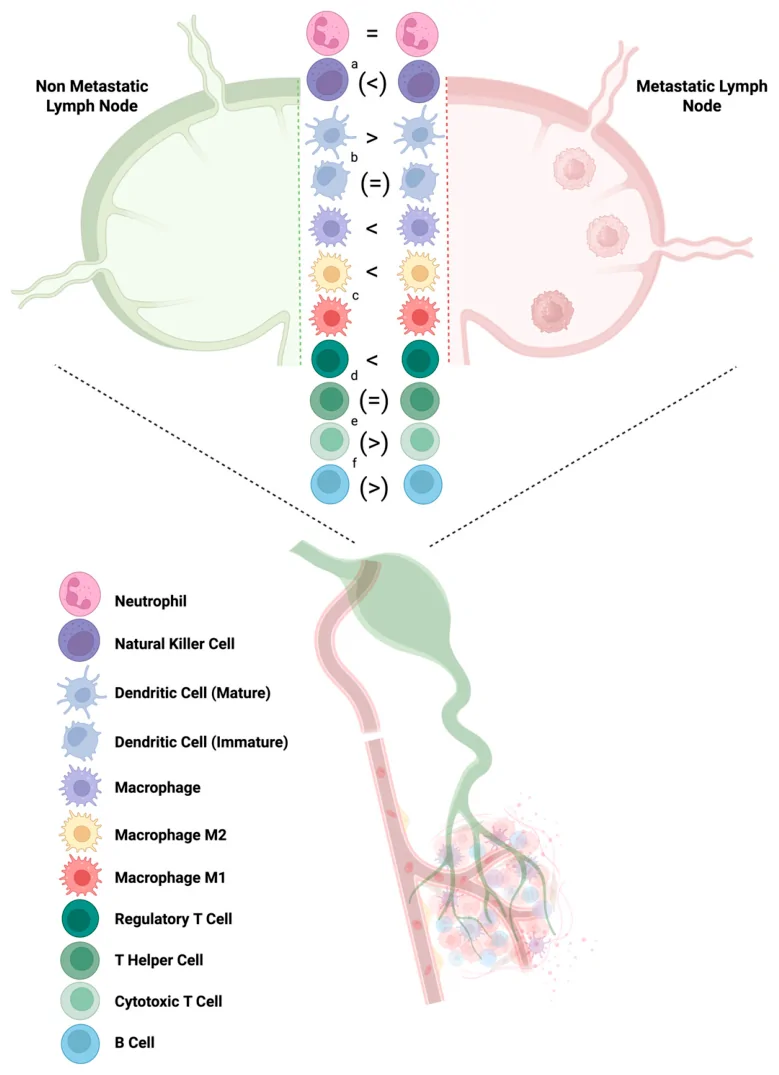
Schematic representation of immune cell infiltration in the metastatic lymph node (LN) and in the non-metastatic LN. This figure summarizes the differential infiltration between the metastatic LN and the non-metastatic LN in terms of immune cell density. “>” meaning “higher than”, “<“ meaning “lower than”, “=“ meaning “comparable” between the two environments. ^a^ **Natural killer cell**. Results are inconsistent but mainly express that the density is higher in the early metastatic LN. ^b^ **Immature dendritic cells**. Results are inconsistent, but the majority of authors report that the density is comparable between the two environments. ^c^ **Macrophages M1**. Results are inconsistent. ^d^ **T helper cells**. Results are inconsistent, but the majority of authors report that the density is comparable between the two environments. ^e^ **Cytotoxic T cells**. Results are inconsistent, but the majority of authors report that the density is lower in metastatic LN. ^f^ **B cells**. Results are inconsistent, but the majority of authors report that the density is lower in metastatic LN.

Regarding the role of DCs in the response to HNSCC, the investigation of the current literature has provided us with a more precise understanding of the impact of antigen presentation. While there are some discrepancies in the findings we have gathered, the prevailing trend in most reports underscores the protective role of DCs [22,33]. A schematic representation of immune cell infiltration in the metastatic LN and in the non-metastatic LN is represented in Figure 3. 

The significance of DCs has been more convincingly highlighted and correlated with improved survival rates and with better control over lymph node metastases [22,25,33,34]. These results are significant since they point out the first immunological pathway involved in the defense against tumor cells. By happening in the lymph node and being attributed to dendritic cells, energy must be spent on the recruitment and maturation of DCs in lymph nodes. Furthermore, this aligns with the hypothesis that preserving lymph nodes during cancer treatment may result in an enhanced antitumor response through the recruitment and activation of immune cells. Accordingly, in surgical or radiotherapeutic practice, a new perspective may be considered: in selected cases of localized HNSCC with an indication for adjuvant or neoadjuvant immunotherapy, preserving regional lymph nodes—or at least deferring their treatment—could be discussed in order to maintain their immunological function as priming sites. However, such an approach must remain highly selective, balancing the potential immunological benefit with the oncologic risk of nodal metastasis [43].

In the context of antigen presentation, our findings highlight the protective role of subcapsular sinus macrophages in lymph node metastases. Specifically, we observed a significant contribution of CD169-positive macrophages, confirming the key role of antigen presentation in the antitumor immune response [26]. In clinical practice, this opens two potential avenues: (i) performing CD169 immunostaining on lymph node specimens could help stratify patients according to their “protective state”, and (ii) patients with low CD169 expression may benefit from treatment intensification (e.g., adjuvant immunotherapy or more extensive neck dissection), whereas those with high CD169 expression could be considered for less aggressive management or even treatment de-escalation.

Moreover, macrophages, along with DCs, NK cells, and neutrophils, play pivotal roles in innate immunity, extending beyond their conventional function of antigen presentation. Cells comprising the innate immune response exhibit a dual role in disease progression. On the one hand, they act as the first responders, recognizing malignant cells and initiating immune responses. On the other hand, these same cells, influenced by regulatory and inhibitory signals, can paradoxically promote tumor progression [49]. TAMs exhibit two distinct phenotypes, where M1 subsets generally contribute to the antitumor response through their pro-inflammatory activities, while M2 subsets can suppress this response through their anti-inflammatory actions [50].

Regarding NK cells, our findings suggest that these cells may play a role in the early immune response against occult tumor dissemination in lymph nodes. The observation of increased NK cell density in clinically negative but pathologically positive nodes supports their involvement in the initial detection of metastases. However, the lack of difference between metastatic and non-metastatic nodes in the other study indicates that this response may be transient, suppressed, or insufficient to prevent nodal progression. Further investigations are needed to clarify whether NK cell activity could serve as an early biomarker of immune engagement or a target for immunomodulation in the pre-metastatic phase [25,37].

In the case of macrophages, some authors identified elevated levels of TAMs in metastatic lymph nodes. However, in HPV+ oropharyngeal SCC, an increased macrophage count surprisingly correlates with better survival outcomes [16]. This observation aligns with the findings developed by Lechien et al., concluding that there is massive recruitment of immune cells in HPV+ HNSCC [51]. Conversely, the understanding of macrophage polarization is intricate, lacking coherence on this front.

Within the innate immune response, the contribution of neutrophils to cancer control was proposed several years ago, though their impact has been overshadowed by the presence of other cell types. There is evidence suggesting that these cells can have a direct cytotoxic effect on tumor cells and may also indirectly induce a specific T cell response [35,36].

As a result, although the analysis of these cells (TAMs, DCs, NK cells, and neutrophils) in lymph nodes holds promise as a prognostic or predictive tool, its integration into clinical decision-making is currently premature. Further investigations are needed to clarify the immunological signatures that could reliably inform treatment intensity, particularly in the context of immunotherapy or nodal preservation strategies. Until then, these immune features should be considered exploratory markers rather than actionable parameters in head and neck oncology.

Innate immunity serves as a pivotal trigger for the adaptive immune response, a crucial defense mechanism against tumor cells. The immune infiltrate components associated with the adaptive response are numerous, with some exerting a beneficial role in the antitumor response, while others may promote tumor growth [52].

Tregs, due to their ability to potentiate immune suppression by dampening T cell activation, have been extensively studied for their impact. Contrary to our expectations, an increased density of Tregs in the lymph node microenvironment has been suspected to be associated with better OS rates. The results emphasize that even though they are not significant, there is still a trend that needs to be considered in the role of Tregs. They are consistent with those obtained by Furgiuele et al. in their study of the TME [53]. The potential explanation lies in the fact that a high infiltration of Tregs may reduce the pro-tumoral impact of inflammatory cells [54]. There may be unidentified pathways explaining the aforementioned inconsistencies. The gut microbiome could be one of the environmental factors that might influence the TME and account for the different behaviors observed between cancers localized along the gastrointestinal tract [55,56,57]. To enhance comprehension of these elusive pathways, spatial analysis of immune cells and their correlation with the inflammatory microenvironment (hot versus cold tumors) are imperative. In several solid cancers, there is a belief that targeting the elimination of Tregs can activate tumor effector cells, thereby enhancing the efficacy of immunotherapy [58]. 

Furthermore, the crucial role of Th cells in establishing an antitumor response has been extensively studied, particularly in the context of immunotherapy, which has revolutionized cancer treatment [59]. It is strongly posited that Th cells play a foundational role in promoting antitumor immunity by providing support to cytotoxic T cells [59]. Our review highlights the potential impairment in Th cell expression in lymph node metastases, a phenomenon correlated with diminished OS rates. Similarly, cytotoxic T cells, along with Th cells, constitute essential components of the adaptive immune system, working together to eradicate aberrant cells and orchestrate an effective immune response [60,61,62]. In lymph nodes of HNSCC patients, the density of cytotoxic T cells has been mostly associated with improved OS and reduced lymph node metastases [11,21,38,43,45].

Contrary to the well-established role of T cells in tumor cells, less is known about tumor-infiltrating B cells. B cells possess the ability to produce antibodies, form antigen-antibody complexes, present antigens through their antigen presentation capabilities, and secrete cytokines [63]. Based on current research, B cells in the TME are predominantly associated with tertiary lymphoid structures (TLSs), and the presence of mature TLSs with a high density of B cells is generally linked to better clinical outcomes [64]. However, the role of B cells and the presence of TLSs remain controversial in HNSCC, even though the densities of B cells and B/T^cytotoxic^ cell interactions positively impact OS [65].

Based on this comprehensive review, the analysis regarding the association between B lymphocytes and tumor progression remains inconclusive. However, emerging evidence suggests that nodal metastases exhibit a significant reduction in B lymphocyte levels [43,46]. Moreover, with regard to survival outcomes, B lymphocytes appear to confer a beneficial role [44].

As a result, the interplay between adaptive immune cells in lymph nodes is complex and suggests the potential for developing immune-based scoring systems. Rather than the evaluation of individual cell types, we believe that the establishment of a multiparametric immunoscore could offer a more robust reflection of the adaptive immune system behavior within the nodal environment.

Therefore, efforts should be directed towards regulating adaptive immunity within the lymph nodes in HNSCC. Promoting antigen presentation, as mentioned above, provides insights to enhance the antitumor response. However, targeting the adaptive immune system in lymph nodes also appears to be an alternative approach for developing therapeutic strategies. Furthermore, understanding the immunological profile of lymph nodes, especially pre-metastatic nodes, holds the potential to achieve more precise staging with prognostic implications that surpass the accuracy of currently employed systems.

Finally, the reported findings contribute to a more comprehensive understanding of the antitumor response and the immune landscape within lymph nodes. The lymph node microenvironment is highly enriched with immune cell infiltration, and its effective roles in the antitumor response are, for the most part, coherent with its respective roles in the TME. Similarly, investigations in HPV+ patients have revealed heightened immune cell infiltration in lymph nodes, mirroring the levels observed within the tumor itself.

However, very few studies have compared the density of cell infiltration between primary tumors and lymph nodes, and we are unable to provide evidence confirming whether the immune setting in lymph nodes is enhanced or decreased compared to the primary tumor. Establishing and comparing cell infiltration between these two compartments could offer new approaches in the understanding and treatment of HNSCC.

An additional limitation of this review relates to the NOS variability across studies. Several studies were retrospective, including small patient cohorts, and did not consistently consider important confounding factors such as HPV status, tumor subsite, or treatment history. Therefore, some of the discrepancies observed regarding macrophage polarization, dendritic and natural killer cell recruitment, and T/B cell distribution may be explained by methodological differences rather than true biological variations. For example, a better adjustment for major confounding factors, such as HPV status, could help identify more homogeneous immune profiles and reduce the differences observed across studies. A more consistent stratification according to tumor subsite could improve the accuracy and reproducibility of the findings, as the immune microenvironment is known to vary across different anatomical locations [27]. Finally, systematic evaluation of clinical outcomes, particularly OS and DFS, would strengthen the interpretation of the results.

The considerable heterogeneity reported above has led to limitations in the direct comparison between studies but has also prevented the realization of quantitative data pooling for a potential meta-analysis.

Another limitation of this review is the absence of prospective registration in PROSPERO. Although the protocol was registered in INPLASY, the lack of a prospective registration stands as a methodological weakness. This limitation should be considered during the interpretation of the results.

From a clinical standpoint, the immune landscape of lymph nodes in HNSCC offers promising directions for improving patient care. It may contribute to treatment stratification, help refine surgical decisions, guide immunotherapy use, and support the development of integrative prognostic models. Ultimately, systematic immune profiling of nodal tissues could evolve into a routine adjunct to histopathological analysis, fostering a more personalized oncologic approach. However, the available evidence remains mostly observational and should be interpreted with caution.

## 5. Conclusions

In conclusion, there is emerging evidence suggesting a crucial role of the lymph node immune microenvironment and the immune crosstalk between lymph nodes and HNSCC. The understanding of the physiological pathways underlying this disease and the immune responses involved is still insufficient to provide a clear prognosis and predictive model. Even though many reports have highlighted the critical impact of antigen presentation in the antitumoral response, promising new perspectives are unfolding in this field of research, emphasizing the potential role of different types of immune cells. The low number of available studies, the multitude of cells involved, the complexity of cell interactions, the inability to consider cofactors, and the effort needed to obtain different tissues from the same patient may explain the inconsistent results and the inconvenience of establishing an immunological model able to predict HNSCC prognosis. Future research priorities and consistent methodologies are essential to overcome these limitations. Standardizing lymph node classifications, approaches for immune cell identification and quantification, the assessment of confounding factors, and the evaluation of clinical outcomes would improve the comparability of future studies. These efforts could help develop novel immune-based prognostic tools and support de-escalation and personalized therapeutic strategies in head and neck oncology.

## Figures and Tables

**Table 1:** Studies included in analysis regarding cell investigation.

Authors	Year	Neutrophils	Natural Killer Cells	Dendritic Cells	Macrophages	Regulatory T Cells	T Helper Cells	Cytotoxic T Cells	B Cells	Lymphocytes	Surface Markers
Goncalves et al. [22]	2013			✓		✓					
Hyun Kim et al. [47]	2024									✓	
Joabe Pereira et al. [38]	2014						✓	✓			
Jones et al. [16]	2021				✓			✓		✓	
Kagedal et al. [39]	2020						✓			✓	✓
Kesselring et al. [40]	2011						✓				✓
Kindt et al. [32]	2016			✓							
Kumagai et al. [41]	2010						✓	✓			
Kun Wu et al. [31]	2023			✓	✓		✓	✓	✓		
Letessier et al. [42]	1990						✓	✓		✓	✓
Li et al. [33]	2009			✓							
Li Yicun et al. [37]	2025		✓								
Norouzian et al. [46]	2020								✓		
O’Donnell et al. [34]	2007			✓							
Piersiala et al. [45]	2022					✓					
Pretscher et al. [44]	2009					✓		✓	✓		
Pylaeva et al. [35]	2021	✓									
Pylaeva et al. [36]	2022	✓									
Rahim et al. [43]	2023						✓	✓	✓		
Rubio et al. [24]	2001						✓	✓	✓	✓	
Sakakura et al. [25]	2005		✓	✓							
Topf et al. [26]	2019				✓						✓
Tosi et al. [27]	2022				✓	✓	✓	✓			
Verastegui et al. [11]	2002							✓			
Wang et al. [28]	2022				✓			✓			
Wehrhan et al. [29]	2014				✓						
Zhang et al. [30]	2025			✓	✓				✓	✓	

Abbreviation: ✓ indicates that the parameter was assessed in the corresponding study.

**Table 2:** Innate immune cell infiltration in head and neck squamous cell carcinoma lymph nodes.

Authors	Year	No. and Type of Specimen	Findings
Goncalves et al. [22]	2013	Human Specimens Oral Squamous Cell Carcinoma N = 30 LN (3 Groups) Group 1—LN1: Negative LN—Patients without LN Metastases (N = 10) Group 2—LN2: Negative LN—Patients with LN Metastases (N = 10) Group 3—LM2: Positive LN—Patients with LN Metastases (N = 10)	Dendritic cells DCs (CD1a+) higher in LM2 DCs (CD83+) lower in LM2 CD1a+/CD83+ ratio higher in LM2
Jones et al. [16]	2021	Mice and Human Specimens Breast Cancers, Head and Neck Cancers, Colon Cancers N = 9 Patients (HNSCC)	Macrophages Macrophages are more frequent in M+ LN (than in M− LN)
Kindt et al. [32]	2016	Human Specimens Head and Neck Squamous Cell Carcinoma N = 125 Primary Tumors N = 118 Dysplasia	Dendritic cells DCs are higher in M+ LN compared with M− LN
Kun Wu et al. [31]	2023	Human Specimens Oral Squamous Cell Carcinoma N = 11 Patients Group 1—M^−^ LN (N = 10) Group 2—M^+^ LN (N = 10)	Macrophages and dendritic cells No differences between the distribution of macrophages and DCs in M^+^ and M^−^ LN
Li et al. [33]	2009	Human Specimens Hypopharyngeal, Laryngeal Squamous Cell Carcinoma N = 68 Patients	Dendritic cells Number of DCs (S-100, CD1a, CD83, CD45RO) is higher in M− LN compared with M+ LN DCs (CD1a) are mainly localized in the tumoral compartment in M+ LN DCs (CD83) are mainly localized in the peri-tumoral compartment in M+ LN DCs (S-100, CD1a, and CD83) are associated with better OS
Li et al. [37]	2025	Human Specimens (from TCGA) Head and Neck Squamous Cell Carcinoma N = 198 LN Group 1—cN0 pN0 (N = 140) Group 2—cN0 pN+ (N = 58)	Natural killer cells NK higher in cN0-pN+ than in cN0-pN0
O’Donnell et al. [34]	2007	Human Specimens Oral Squamous Cell Carcinoma N = 63 Primary Tumors, 8 LN	Dendritic cells Number of total DCs and immature DCs is similar in M+ and M− LN Number of mature DCs is higher in uninvolved LN of M+ patients (compared with M− patients) Number of mature DCs in LN is decreased with the distance from primary tumor (in M+ and M− patients)
Pylaeva et al. [35]	2021	Human Specimens Head and Neck Squamous Cell Carcinoma N = 71 Patients	Neutrophils Neutrophils (CD66b^+^) (in B cell areas) in LN is associated with increased B cell proliferative activity Number of neutrophils (in B cell areas) is similar between metastatic and non-metastatic LN Neutrophils (in B cell areas) in LN improves OS
Pylaeva et al. [36]	2022	Human Specimens Head and Neck Squamous Cell Carcinoma N = 121 Patients	Neutrophils Number of neutrophils is similar between metastatic and non-metastatic LN Neutrophils are mostly localized in sinuses (close to T cells) In M+ patients, a high number of neutrophils in LN is associated with poor OS In M− patients, a higher number of neutrophils in LN is associated with better OS
Sakakura et al. [25]	2005	Human Specimens Oral Squamous Cell Carcinoma N = 12 Patients N = 89 LN	Natural killer cells NK cells are similar in sentinel and non-sentinel LN NK cells are similar in positive and negative sentinel LN Dendritic cells Number of immature DCs is higher in sentinel LN (compared with non-sentinel LN) (no difference for mature DCs) No difference in mature and immature DCs between M+ and M− sentinel LN
Topf et al. [26]	2019	Human Specimens Oral and Oropharyngeal Squamous Cell Carcinoma N = 22 Patients	Macrophages M+ LN show lower levels of CD68+ macrophages in subcapsular sinuses, compared to adjacent M− LN
Wang et al. [28]	2022	Human Specimens Oral Squamous Cell Carcinoma N = 18 Patients	Macrophages M1 macrophages are increased, and M2 macrophages are decreased in LN metastases
Wehrhan et al. [29]	2014	Human Specimens Oral Squamous Cell Carcinoma N = 54 LN	Macrophages The macrophage polarization is similar between M− LN and M+ LN M2 macrophages in LN are increased, and M1 macrophages in primary tumors are decreased with higher L, Pn, and T status and grading
Zhang et al. [30]	2025	Human Specimens Oral Squamous Cell Carcinoma N = 68 Patients Group 1—M^−^ LN (N = 68) Group 2—M^+^ LN (N = 68) Group 3—PT (N = 68)	Dendritic cells Proportion of DCs is similar in all groups Macrophages Proportion of macrophages is (3 = 2) > 1

Abbreviations: No, number; M+, metastatic; M−, non-metastatic; LN, lymph node; OS, overall survival; HNSCC, head and neck squamous cell carcinoma; DFS, disease-free survival; NK cells, natural killer lymphocytes; DC, dendritic cell.

**Table 3:** Adaptive immune cells infiltration in head and neck squamous cell carcinoma lymph nodes.

Authors	Year	No. and Type of Specimen	Findings
Goncalves et al. [22]	2013	Human Specimens Oral Squamous Cell Carcinoma N = 30 LN (3 Groups) Group 1—LN1: Negative LN—Patients without LN Metastases (N = 10) Group 2—LN2: Negative LN—Patients with LN Metastases (N = 10) Group 3—LM2: Positive LN—Patients with LN Metastases (N = 10)	Regulatory T Lymphocytes Tregs are higher in LM2 (compared to LN1—LN2)
Hyun Kim et al. [47]	2024	Human Specimens Head and Neck Squamous Cell Carcinoma N = 21 Patients Group 1—M^+^ LN (N = 21) Group 2—PT (N = 21)	Lymphocytes The TIL scores between M^+^ LN and PT are comparable
Joabe Pereira et al. [38]	2014	Human Specimens Oral Squamous Cell Carcinoma N = 50 patients	T Helper Lymphocytes and Cytotoxic T Lymphocytes No difference in the density of CD4^+^ T cells between M^+^ and M^−^ LN CD8^+^/CD4^+^ ratio is higher in M^−^ LN
Jones et al. [16]	2021	Mice and Human Specimens Breast Cancers, Head and Neck Cancers, Colon Cancers N = 9 Patients (HNSCC)	Cytotoxic T Lymphocytes CD8^+^ T cells more frequent in M^−^ LN than in M^+^ LN Lymphocytes Tumor growth in LN induces a reduction in lymphocyte recruitment
Kagedal et al. [39]	2020	Human Specimens Oral Squamous Cell Carcinoma N = 28 patients Analysis of Sentinel Nodes, Non-Sentinel Nodes, Primary Tumors, and PBMC	T Helper Lymphocytes Patients with recurrent OSCC have lower percentage of CD4^+^CD69^+^ T cells in LN Patients with low level of CD4^+^CD69^+^ T cells in LN have worse DFS
Kesselring et al. [40]	2011	Human Specimens Head and Neck Squamous Cell Carcinoma Healthy Control Patients (n = 25), Peripheral Blood HNSCC Patients (n = 25), Tumor Sample (n = 10), M^+^ LN (n = 20)	T Helper Lymphocytes The expression of surface marker CD161 is impaired on tumor-infiltrating Th17 T cells derived from M^+^ LN
Kumagai et al. [41]	2010	Human Specimens Head and Neck Squamous Cell Carcinoma N = 17 Patients	T Helper Lymphocytes and Cytotoxic T Lymphocytes The expression of CD4^+^ and CD8^+^ T cells is similar between M^+^ and M^−^ LN
Kun Wu et al. [31]	2023	Human Specimens Oral Squamous Cell Carcinoma N = 11 Patients Group 1—M^−^ LN (N = 10) Group 2—M^+^ LN (N = 10)	T Helper Lymphocytes CD4^+^ T cells are more frequent in M− LN than in M+ LN Cytotoxic T Lymphocytes and B Lymphocytes No differences between the distribution of B cells and CD8^+^ T cells in M^+^ and M^−^ LN
Letessier et al. [42]	1990	Human Specimens Head and Neck Squamous Cell Carcinoma N = 18 Patients	Cytotoxic T Lymphocytes and T Helper Lymphocytes CD4^+^/CD8^+^ ratio is higher in LN than in blood Lymphocytes Lymphocytes in LN had fewer CD56^+^ and CD11b^+^ cells than blood lymphocytes No difference between M^+^ and M^−^ LN lymphocytes
Norouzian et al. [46]	2020	Human Specimens Head and Neck Squamous Cell Carcinoma N = 32 Patients	B Lymphocytes No differences between the distribution of B cells in M^+^ and M^−^ LN (in laryngeal SCC) B cells are more frequent in M^−^ LN (in tongue SCC)
Piersiala et al. [45]	2022	Human Specimens Oral Squamous Cell Carcinoma N = 23 Patients	Regulatory T Lymphocytes The number of Tregs is higher in TDLN than in non-TDLN The number of Tregs is higher in M+ LN compared with M− LN
Pretscher et al. [44]	2009	Human Specimens Head and Neck Squamous Cell Carcinoma N = 33 Patients	Regulatory T Lymphocytes There is a non-significant trend for improved DFS rates for patients with an increased infiltration of Tregs in M+ LN Cytotoxic T Lymphocytes CD8^+^ T cells are more frequent in M^−^ LN than in M^+^ LN Number of CD8^+^ T cells in M^+^ LN is associated with better DFS B Lymphocytes Number of CD20^+^ B cells is higher in M^+^ LN compared to primary tumor Number of CD20^+^ B cells in M^+^ LN is associated with better DFS rates
Rahim et al. [43]	2023	Human Specimens Head and Neck Squamous Cell Carcinoma N = 10 Patients	T Helper Lymphocytes CD4^+^ T cells are more frequent in M− LN than in M+ LN Cytotoxic T Lymphocytes The density of CD8^+^ T cells is similar between M^+^ and M^−^ LN CD8^+^ Tpex cells are more frequent in M^−^ LN than in primary tumors (contrary to CD8^+^ Tex cells) The reservoir of Tpex cells stands in M^−^ LN and plays an important role in immune response Tpex are almost absent in M^+^ LN, and Tex are more abundant in M^+^ LN B Lymphocytes B cells are more frequent in M^−^ LN than in M^+^ LN
Rubio et al. [24]	2001	Human Specimens Head and Neck Squamous Cell Carcinoma N = 102 LN	T Helper Lymphocytes Number of CD4^+^ T cells is greater in control LN than in pre-metastatic LN Number of CD4^+^CD69^+^ T cells is comparable between both groups Cytotoxic T Lymphocytes CD8^+^ T cells are more frequent in control LN than in pre-metastatic LN Lymphocytes T cells in control LN are naïve and are less frequent in pre-metastatic LN The number of CD69^+^ lymphocytes is comparable between control and pre-metastatic LN Pre-metastatic LN exhibits follicles with four layers of cell populations (B cells in the germinal center, T cells in the centrogerminal layer, B cells in the mantle, T cells outside the mantle) In pre-metastatic LN, T cells from the centrogerminal layer are CD69^+^, and T cells in the outer zone of the mantle are CD69^−^ In pre-metastatic LN, B cells from the central zone are CD69^−^, and B cells from the mantle are CD69^+^ B Lymphocytes Number of B cells is lower in control LN than in pre-metastatic LN
Verastegui et al. [11]	2002	Human Specimens Head and Neck Squamous Cell Carcinoma N = 30 Patients	Cytotoxic T Lymphocytes Number of CD8^+^ T cells M^+^ LN is lower than in control LN Proximal LN show a decrease in CD8^+^ T cells (compared to the distal ones)
Wang et al. [28]	2022	Human Specimens Oral Squamous Cell Carcinoma N = 18 Patients	Cytotoxic T Lymphocytes CD8^+^ T cells are increased, and Tex cells are decreased in M^+^ LN
Zhang et al. [30]	2025	Human Specimens Oral Squamous Cell Carcinoma N = 68 Patients Group 1—M^−^ LN (N = 68) Group 2—M^+^ LN (N = 68) Group 3—PT (N = 68)	B Lymphocytes Proportion (%) of B cells are 1 > 2 > 3 T Lymphocytes Proportion (%) of T cells are (1 = 2) > 3

Abbreviations: No, number; M+. metastatic; M−, non-metastatic; LN, lymph node; DFS, disease-free survival; HNSCC, head and neck squamous cell carcinoma; SCC, squamous cell carcinoma; PBMC, peripheral blood mononuclear cell; OSCC, oral squamous cell carcinoma; Tpex, precursor exhausted cells; Tex, exhausted cells; Tregs, regulatory T cells.

**Table 4:** Immune cell infiltration in HPV-associated oropharyngeal cancer lymph nodes.

Authors	Year	No. and Type of Specimen	Findings
Tosi et al. [27]	2022	Human Specimens Oropharyngeal Squamous Cell Carcinoma N = 39 Patients	Macrophages Higher immune cell density in metastases from HPV-positive patients (including CD68/CD163 macrophages) (compared to HPV-negative patients) Macrophages are closer to tumor cells in metastases from HPV-positive patients In HPV-positive patient metastases, higher densities of CD68/CD163 macrophages are associated with better DFS In HPV-negative patient metastases, higher densities of CD163 macrophages are associated with shorter DFS Regulatory T Lymphocytes Tregs are closer to tumor cells in M+ LN from HPV-positive patients T Helper Lymphocytes In HPV-positive patient M+ LN and CD4^+^ T cells are associated with better DFS Cytotoxic T Lymphocytes Number of CD8+ T cells is higher in M^+^ LN from HPV-positive patients CD8^+^ T cells and macrophages are closer to tumor cells in M^+^ LN from HPV-positive patients CD8^+^ T cells are associated with better DFS (in M^+^ LN from HPV-positive patients)

Abbreviations: No, number; M+, metastatic; M−, non-metastatic; LN, lymph node; DFS, disease-free survival; Tregs, regulatory T cells.

## Data Availability

The datasets used and/or analyzed during the current study are available from the corresponding author upon reasonable request.

## Supplementary Materials

The following supporting information can be downloaded at: https://www.mdpi.com/article/10.3390/jpm16090444/s1, Supplementary Table S1: Bias Assessment. The risk of bias summary plot shows the risk for each of the included studies. The green, yellow, and red point represent low, unclear, and high risk, respectively. Supplementary Table S2: Surface marker immune cells in head and neck squamous cell carcinoma lymph nodes. Supplementary Table S3: PRISMA 2020 checklist.

## Abbreviations

The following abbreviations are used in this manuscript:

APC	Antigen-Presenting Cell
DC	Dendritic Cell
DFS	Disease-Free Survival
HNSCC	Head and Neck Squamous Cell Carcinoma
LN	Lymph Node
M−	Non-Metastatic
M+	Metastatic
NK	Natural Killer
No	Number
NOS	Newcastle–Ottawa Scale
OS	Overall Survival
OSCC	Oral Squamous Cell Carcinoma
PBMC	Peripheral Blood Mononuclear Cell
SCC	Squamous Cell Carcinoma
SSM	Subcapsular Sinus Macrophage
TAM	Tumor-Associated Macrophage
TDLN	Tumor-Draining Lymph Node
Th cell	T Helper Cell
TLS	Tertiary Lymphoid Structure
TME	Tumor Microenvironment
Treg	Regulatory T Cell
